# Efficacy of neuroendoscopy-assisted surgery in the treatment of chronic subdural hematoma: a meta-analysis

**DOI:** 10.1186/s41016-024-00380-5

**Published:** 2024-10-09

**Authors:** Hou-Qiang Liu, Xue Bai, Fang-Ling Xiong, Ming-Ming Gao, Huai-Bing Zhang, Bao-Hua Liu

**Affiliations:** 1 Department of Neurosurgery, the Affiliated Suqian Hospital of Xuzhou Medical University Or Suqian Hospital of Nanjing Drum Tower Hospital Group, SuqianJiangsu Province, 223800 China; 2 Affiliated Hospital of Xuzhou Medical University, Xuzhou, Jiangsu Province 221000 China

**Keywords:** Neuroendoscopy, Chronic subdural hematoma, Meta

## Abstract

**Background:**

Chronic subdural hematoma (CSDH) is one of the most common diseases in neurosurgery. It is the result of chronic intracranial hemorrhage that converges between the dura mater and arachnoid three weeks after externally injuring the head. Chronic subdural hematomas are a common complication in neurosurgery. With the gradual increase in the amount of hematoma, the surrounding brain tissue is pushed and compressed, resulting in corresponding clinical symptoms and signs. It is reported that the overall incidence rate of CSDH is 1.72 to 20.6 per 100,000 people every year, and the incidence rate of the elderly is particularly high.

**Methods:**

The computer retrieves eight databases to obtain controlled trials at home and abroad on the effects of neuroendoscopy-assisted surgery in patients with chronic subdural hematoma. After a rigorous literature quality evaluation, data analysis was performed using RevMan 5.3 software.

**Results:**

Twenty studies were ultimately included in this meta-analysis. Seventeen studies reported the Recurrence rate of the test group and the control group, which was significantly lower (OR 0.27; 95% Cl 0.18, 0.38; *P* < 0.01) than the control group, Recovery rate (OR 1.18; 95% Cl 1.01, 1.38; *P* = 0.03), Total effective rate (OR 1.11; 95% Cl 1.04, 1.17; *P* < 0.01), Operative time (SMD 15.78; 95% Cl 9.69, 21.86; *P* < 0.01), Hospital stay (SMD − 1.66; 95% Cl − 2.17, − 1.14; *P* < 0.01) and Complications (OR 0.48; 95% Cl 0.30, 0.78; *P* < 0.01).

**Conclusion:**

The results of this study suggest that neuroendoscopy-assisted surgery may be effective in patients with chronic subdural hematoma, as evidenced by recurrence rate, recovery rate, total effective rate, operative time, hospital stay, complications, and the above conclusions need to be verified by more high-quality studies.

## Background

Chronic subdural hematoma (CSDH) is one of the most common diseases in neurosurgery. An externally injured patient’s head is inflicted with chronic intracranial hemorrhage that occurs 3 weeks after externally inflicting the injury on the dura mater and arachnoid. With the gradual increase in the amount of hematoma, the surrounding brain tissue is pushed and compressed, resulting in corresponding clinical symptoms and signs [1]. The manifestations of CSDH are diverse, mainly due to the immediate compression of the intracranial area due to hematoma expansion. The common symptoms of this disease include headache, epilepsy, changes in mental state, weakness, sensory disorders, dysarthria, gait disorders, nausea and vomiting, stroke, and coma [2]. It is reported that the overall incidence rate of CSDH is 1.72 to 20.6 per 100,000 people every year, and the incidence rate of the elderly is particularly high [3]. With the aging population and the increasing use of anticoagulants and antiplatelet drugs, as well as the development of imaging, CSDH has become increasingly common worldwide. It is estimated that by 2025, it may become the most common neurosurgery disease [4].

Although chronic subdural hematoma is a common disease in neurosurgery, its treatment and management are still controversial. Regarding the treatment of CSDH, traditional treatment methods mainly include conservative (observation or medication) treatment and surgical treatment, and the specific treatment method mainly depends on the patient’s hematoma volume, symptoms, and signs; patients with small amount of hematoma and mild symptoms generally choose conservative management, while patients with large amount of hematoma and symptoms generally use surgical treatment represented by drilling and drainage. These traditional treatment methods are considered to be simple and safe. However, regardless of the treatment method, CSDH patients have a certain recurrence rate after treatment. According to literature reports, the recurrence rate can be as high as 10–30% [5]. After the recurrence of hematoma, many patients require multiple hospitalizations or even surgeries, leading to more complications. There are many pathological factors that lead to the recurrence of CSDH after treatment, including inflammation around the hematoma and the newly formed high permeability capillaries in the outer membrane of the hematoma confirmed by histology [6–8]. What complicates the management of CSDH patients is that the main incidence group of CSDH is the elderly, who often have multiple underlying diseases. Therefore, many of them require the use of antiplatelet and anticoagulant drugs. According to statistics, the total incidence rate of CSDH is 13.5/100,000 people per year, but among people over 65 years old, the incidence rate of CSDH has increased three times, approaching 58.1/100,000 people per year. With the aging population, the challenges faced by treating and managing CSDH patients are also increasing, and we may need a more suitable treatment method to reduce the risk of CSDH recurrence.

Although Atorvastatin monotherapy and middle meningeal artery embolization are reported to be effective [9], surgical removal of hematoma is still a consensus in the treatment of chronic subdural hematoma patients with neurological symptoms [10, 11]. Chronic subdural hematomas are commonly treated with burr hole drainage (BHC) with closed drainage systems [12], while other surgical methods include endoscopic treatment, Fried Dough Twists drill drainage, and craniotomy. Endoscopic techniques, including rigid neuroendoscopy and soft neuroendoscopy, have been used for the treatment of chronic subdural hematoma for over 20 years [13]. In recent years, there have been studies suggesting that neuroendoscopy is more effective than drilling and drainage. Comparative studies between rigid neuroendoscopy and drilling and drainage have shown that rigid neuroendoscopy is superior to drilling and drainage in reducing recurrence and complication rates, thereby improving patient prognosis [14]. Thus, we conducted a meta-analysis to examine the effect of neuroendoscopy-assisted surgery in patients with chronic subdural hematoma.

## Materials and methods

### Selection of studies

Patients with chronic subdural hematoma.

### Selection of participants

Patients with chronic subdural hematoma.

### Types of interventions

The intervention group received neuroendoscopy-assisted surgery in the treatment of patients with chronic subdural hematoma, and the control group received burr hole drainage in the treatment of patients with chronic subdural hematoma. In these meta-analyses, there was no statistically significant difference (*P* > 0.05) in the age distribution, hematoma volume, and bilateral hematoma rate between the two groups of patients in all studies, indicating comparability.

### Types of outcome measures

Research suggests that the following tools can be used to assess the effectiveness of neuroendoscopy-assisted surgery in patients with chronic subdural hematomas: ①recurrence rate; ②recovery rate; ③ total effective rate; ④ operative time; ⑤hospital stay; ⑥ complications. In this research, we use the Newcastle-Ottawa Scale (NOS) to determine the quality of individual studies and to compare the quality of studies included in a meta-analysis. The literature included in this study evaluated outcome measures using at least one of the above scales.

### Search strategy

The databases Cochrane Library, PubMed, Embase, Web of Science, CNKI, China Biomedical Literature Database (CBM), VIP, and WanFang are accessed by the computer for the search terms “neuroendoscopy” and “chronic subdural hematoma”. The search time was from the establishment of the library until February 2023. The specific steps of the literature search are (1) search for relevant documents in the Chinese and English databases, read the title, abstract, and Keywords to further identify the search terms for this study; (2) The English database search used “MeSH Terms” to identify the subject terms, searched using a combination of subject words and keywords.

### Data extraction and quality assessment

Following the initial screening of the abstract, two researchers independently read the full text and evaluated the literature screening results. Exchange screening results, discuss dissenting literature, or consult a third researcher until the results are agreed upon. The information extracted from the data includes basic information about the literature, type of study, study object, sample size, intervention content, outcome measures, etc.

### Statistical analysis

This meta-analysis was conducted by using Review Manager (RevMan). Effects are combined: The outcome measures in this study were all measured data, and the tools used to evaluate are different. There are differences between scores, therefore, the standardized mean difference is used (standardized mean difference, SMD) and 95% letters to the zone (confidence interval, CI) As an indicator of effect. (2) Heterogeneity test: chi-square tests are used to determine whether there is heterogeneity between studies, if *P* > 0.1, *I* ^2 ^< 50%, the included studies were said to be more homogeneous, proceed with a fixed-effects model meta-analysis; if *P* < 0.1, *I* ^2^ >  = 50%, heterogeneity was indicated in the included studies, analyze heterogeneous sources, if there is no clinical heterogeneity, a random-effects model is used meta-analyses. Furthermore, possible differences in qualitative factors were subgroup analyzed.

## Results

### Search results

**Fig. 1:**
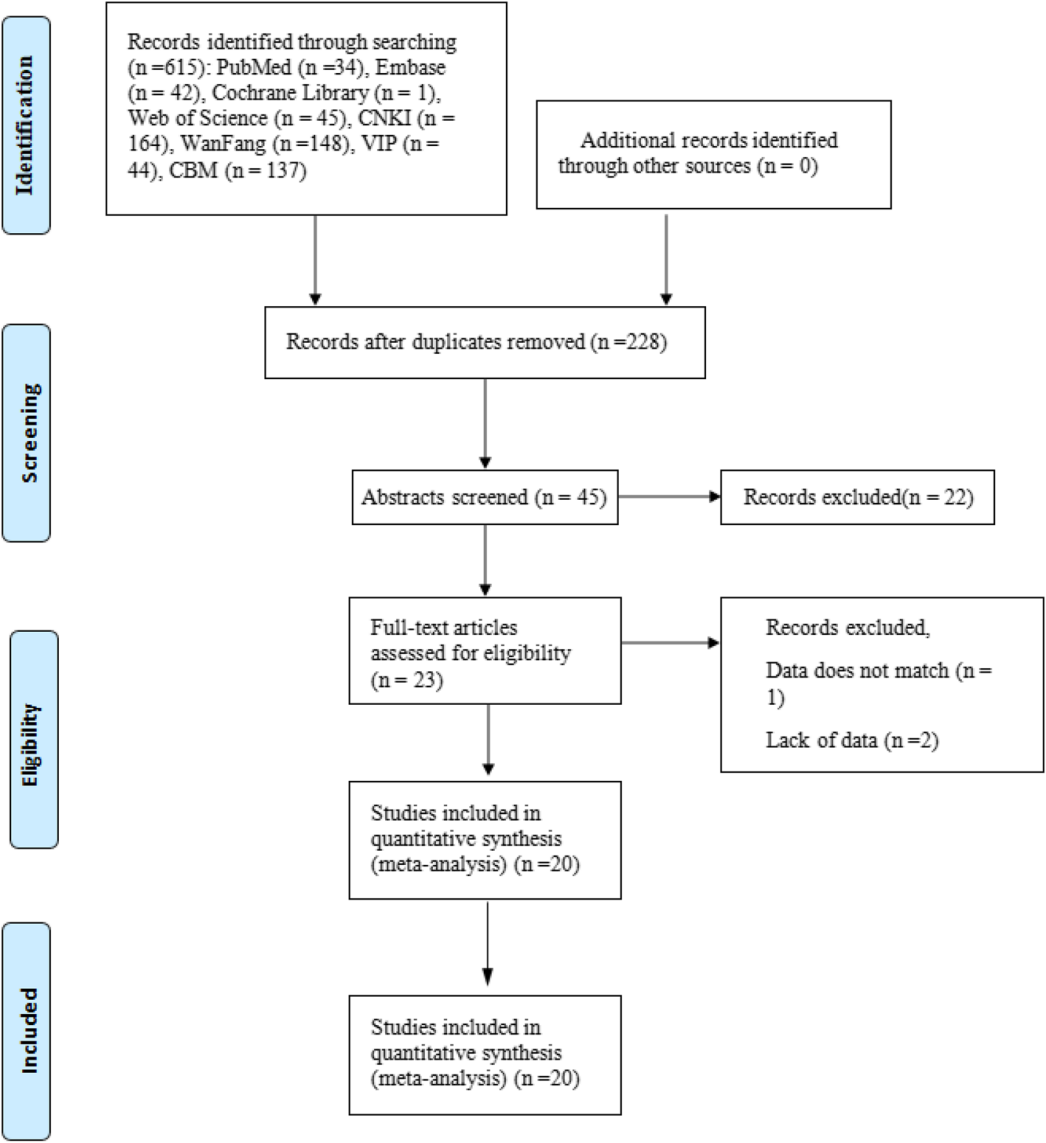
Flow chart

Based on the search strategy, 615 references were identified. After excluding duplicate studies, 45 studies were scanned based on abstract and title. Then, 23 articles were evaluated in full text. After full-text evaluation, 3 records were excluded for the following reasons: data mismatch (*n* = 1) and missing data (*n* = 2). Ultimately, 20 studies [14–33] were included in this meta-analysis (Table 1). The PRISMA statement flow chart shows this process (Fig. 1).

**Table 1:** The basic characteristics of the included studies

Study (ref.)	Sample size (*T*/*C*)	Man /woman	Age (years)(*T*/*C*)	Hematoma volume(ml)	*T*	*C*	Main outcomes
Wu, 2023	46/46	53/39	67.42 ± 4.61/67.45 ± 4.60	None	ND	BH	①②③④⑥
Ma, 2023	48/32	67/43	64.12 ± 7.40/63.58 ± 2.93	42.33 ± 91.11/81.25 ± 4.94	ND	BH	①④⑤⑥
Fang, 2022	41/83	100/24	59.67–68.87/61.26–61.28	109.31–130.03/106.30–128.64	ND	BH	①
Chen, 2022	28/35	34/29	60.25 ± 4.13/60.73 ± 4.14	91.17 ± 7.51/91.69 ± 7.53	ND	BH	①②③④⑤⑥
Chu, 2021	34/34	45/23	63.57 ± 2.94/63.76 ± 3.22	81.26 ± 4.93/81.46 ± 4.98	ND	BH	③④⑤⑥
Deng, 2021	39/39	48/30	73.89 ± 5.01/72.59 ± 4.86	169.54 ± 9.86/165.73 ± 9.51	ND	BH	①②③④⑤⑥
Du, 2020	45/49	59/35	73.2 ± 5.5/70.6 ± 6.1	96.8 ± 19.2/104.3 ± 21.3	ND	BH	①③④
Dai, 2019	52/64	62/54	48.52 ± 6.25	None	ND	BH	①
Guan, 2019	31/126	94/63	68.3(60–81)/71.4(61.3–87.5)	109.3(30.4–167.9)/114.6 (35.2–157.5)	ND	BH	①④⑤⑥
Zhang, 2018	42/31	54/19	74.3(67–91)/75.1(58–95)	30.3–200.2/37.6–171.5	ND	BH	①
Shi, 2018	30/30	None	51.5/53.2	None	ND	BH	①⑥
An, 2018	52/54	82/24	68.75 ± 8.19/66.78 ± 8.71	82.46 ± 14.96/82.69 ± 14.44	ND	BH	①②④⑤
Chen, 2018	40/25	51/14	58.13 ± 9.71/55.52 ± 8.07	32.84 ± 7.02	ND	BH	①④⑤⑥
Hu, 2018	34/34	36/32	61.8 ± 4.1/62.6 ± 3.9	None	ND	BH	④⑥
Jiao, 2018	60/60	None	None	None	ND	BH	①②④⑤
Yan, 2017	24/52	51/25	66.00 ± 6.89/66.38 ± 9.35	None	ND	BH	①④⑤
Zhu, 2017	74/87	141/20	70.47 ± 11.73/68.57 ± 11.27	121.76 ± 32.24/119.14 ± 37.85	ND	BH	①④⑤
Kunting, 2022	13/216	169/60	71.06 ± 15.78/71.23 ± 9.89	None	ND	BH	①④
Yadav, 2020	68/68	84/52	45–79	None	ND	BH	⑥
Amano, 2021	97/283	243/137	77.1 ± 10.3/76.8 ± 10.5	None	ND	BH	①④⑥

*T* trial group, *C* control group, *ND* neuroendoscopy, *BH* Burr-hole

① Recurrence rate, ②Recovery rate, ③Total effective rate, ④Operative time, ⑤Hospital stay, ⑥Complications

### Total effective rate

**Fig. 2:**
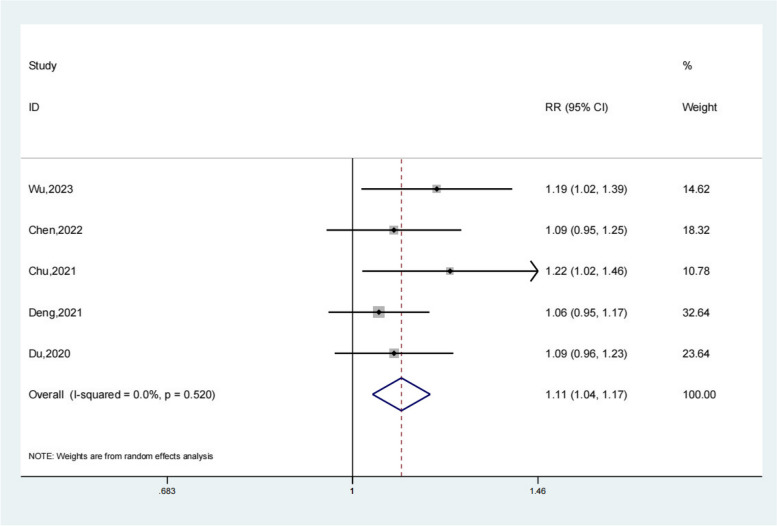
Forest illustration of the total effective rate

**Fig. 3:**
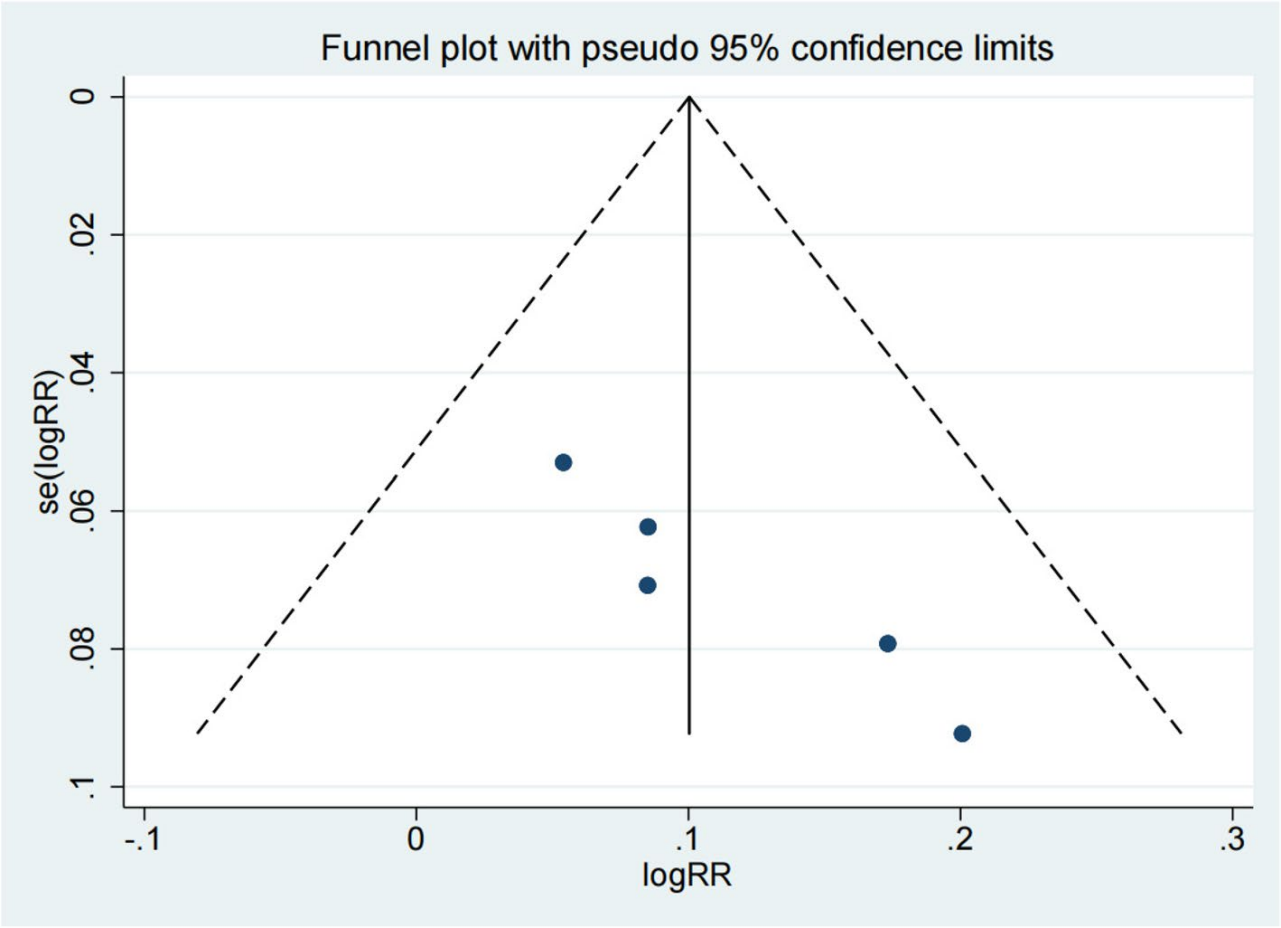
Funnel plot of the total effective rate

Five studies reported the Total effective rate of the test group and the control group. Meta-analysis showed that the Total effective rate of the test group was significantly higher (OR 1.11; 95% Cl 1.04,1.17; *P* < 0.01, Fig. 2) than the control group. The funnel plot is relatively symmetrical (Fig. 3). Compared with the control group, neuroendoscopy-assisted surgery in the treatment of patients with chronic subdural hematoma increases the level of total effective rate.

### Operative time

**Fig. 4:**
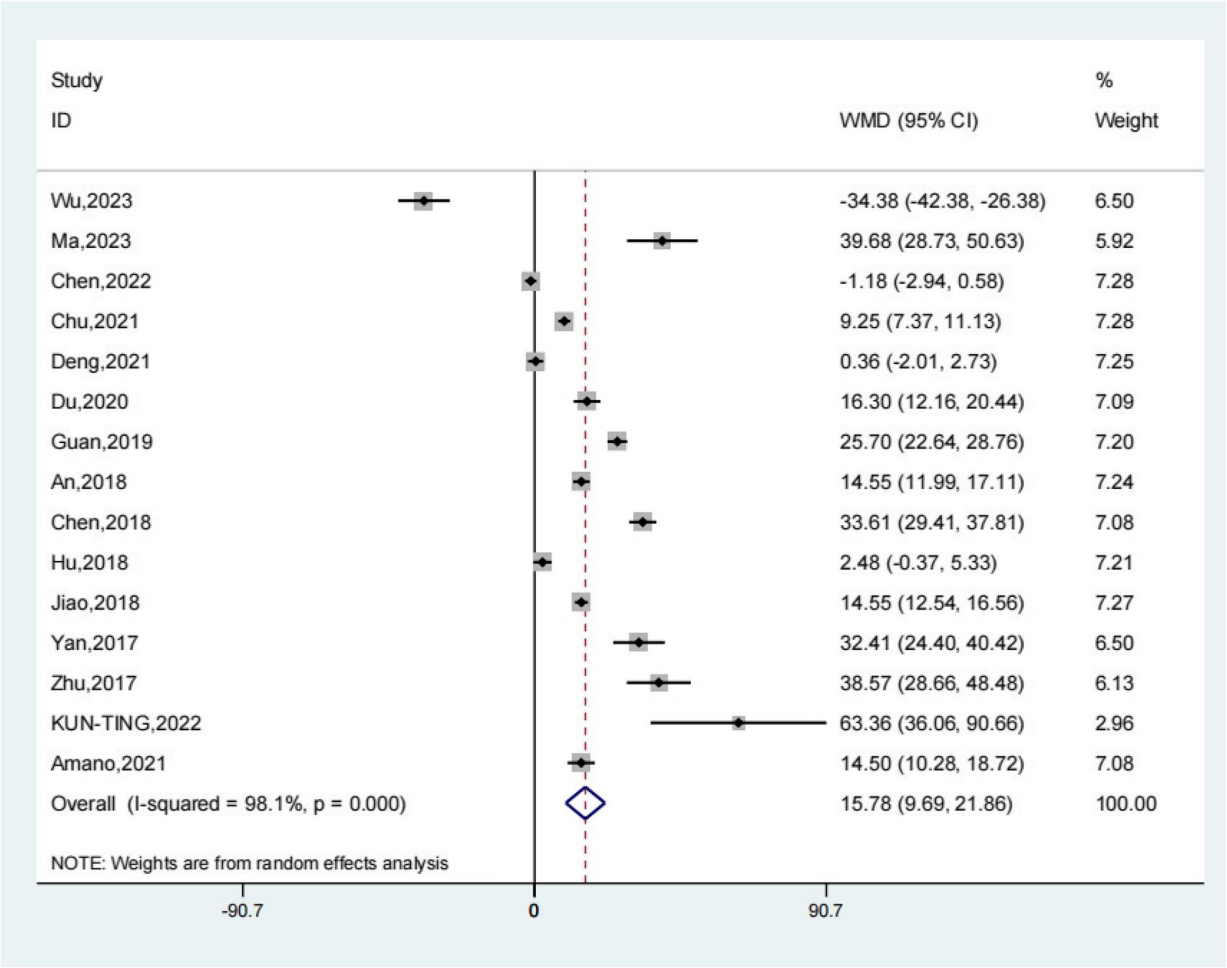
Forest illustration of the operative time

Fifteen studies reported the operative time of the test group and the control group. Meta-analysis showed that the operative time of the test group was significantly higher (SMD 15.78; 95% Cl 9.69, 21.86; *P* < 0.01, Fig. 4) than the control group. Compared with the control group, neuroendoscopy-assisted surgery in the treatment of patients with chronic subdural hematoma increases the level of operative time.

### Hospital stay

**Fig. 5:**
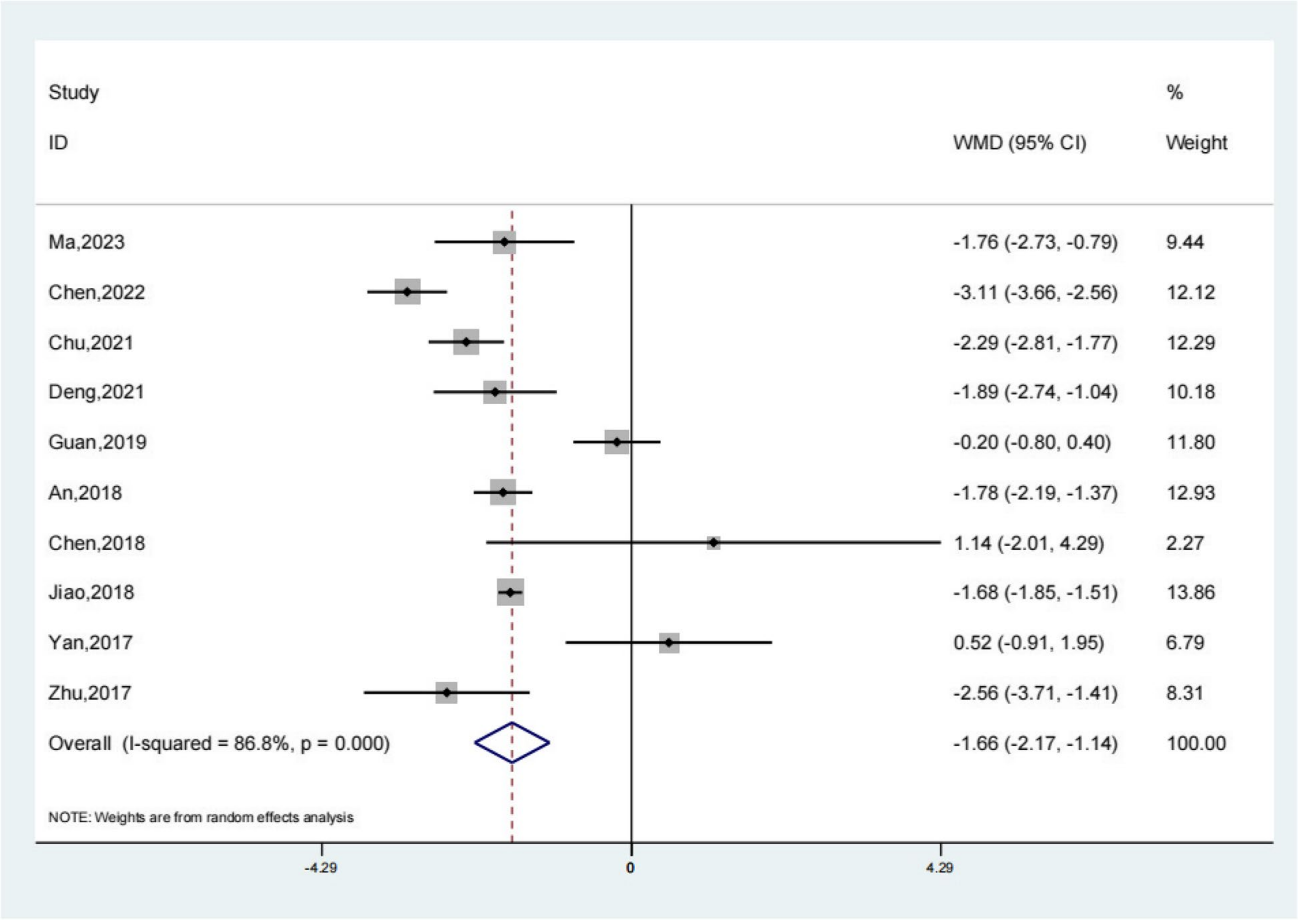
Forest illustration of the hospital stay

Ten studies reported the hospital stay of the test group and the control group. Meta-analysis showed that the hospital stay of the test group was significantly lower (SMD − 1.66; 95% Cl − 2.17, − 1.14; *P* < 0.01, Fig. 5) than the control group. Compared with the control group, neuroendoscopy-assisted surgery in the treatment of patients with chronic subdural hematoma decreases the level of hospital stay.

### Complications

**Fig. 6:**
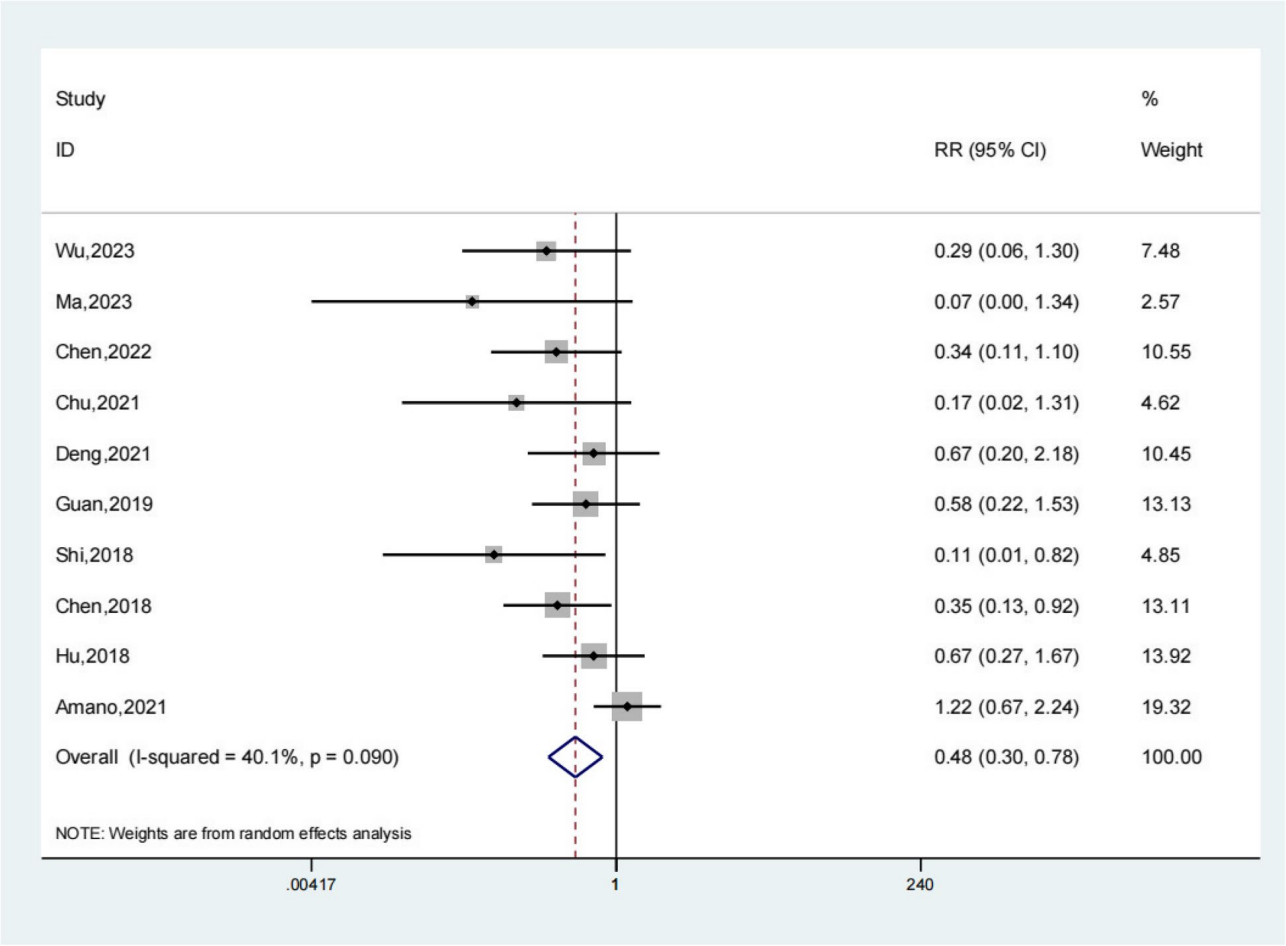
Forest illustration of the complications

Complications are defined as intracranial gas accumulation, residual hematoma, intracerebral hematoma, brain contusion, intracranial infection, and hematoma recurrence. Ten studies reported the complications of the test group and the control group. Meta-analysis showed that the complications of the test group were significantly lower (OR 0.48; 95% Cl 0.30, 0.78; *P* < 0.01, Fig. 6) than the control group. Compared with the control group, neuroendoscopy-assisted surgery in the treatment of patients with chronic subdural hematoma decreases the level of Complications.

### Recovery rate

**Fig. 7:**
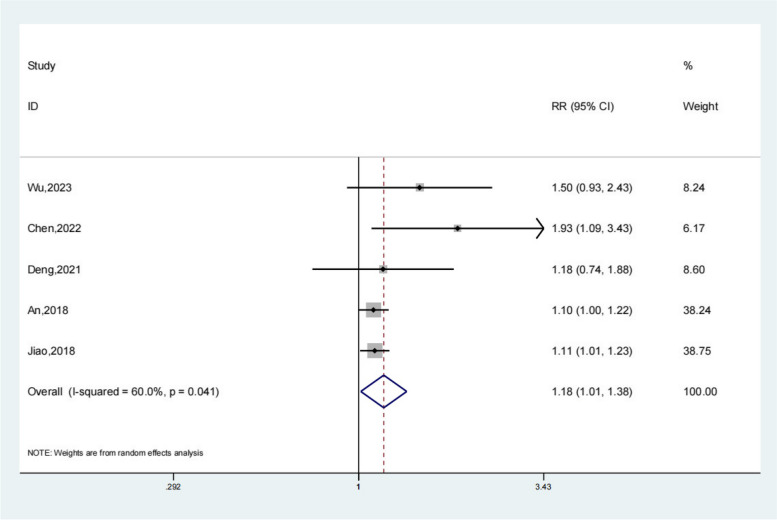
Forest illustration of the recovery rate

**Fig. 8:**
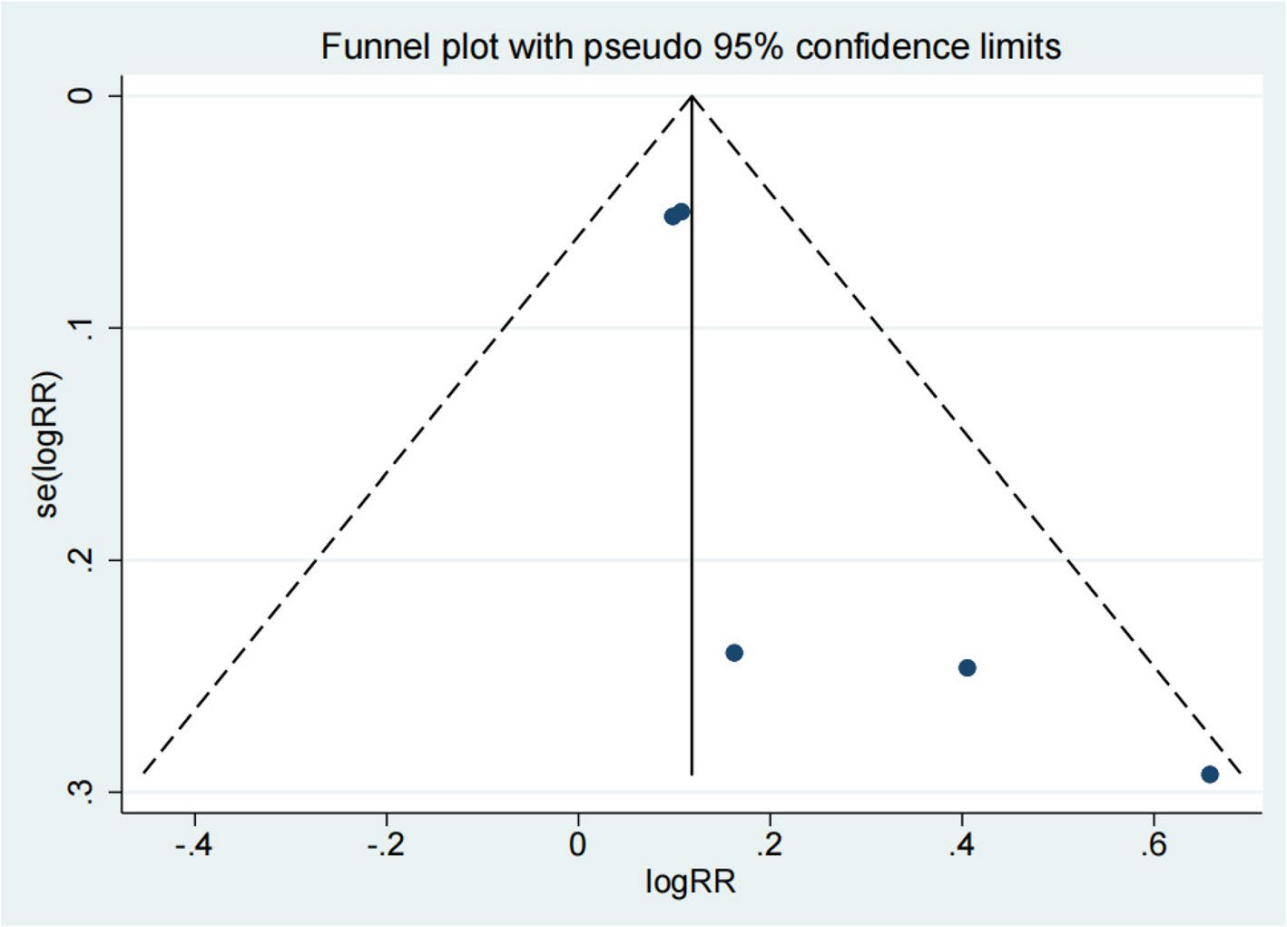
Funnel plot of the recovery rate

Five studies reported the recovery rate of the test group and the control group. Meta-analysis showed that the recovery rate of the test group was significantly higher (OR 1.18; 95% Cl 1.01,1.38; *P* = 0.03, Fig. 7) than the control group. The funnel plot is relatively symmetrical (Fig. 8). Compared with the control group, neuroendoscopy-assisted surgery in the treatment of patients with chronic subdural hematoma increases the level of recovery rate.

### Recurrence rate

**Fig. 9:**
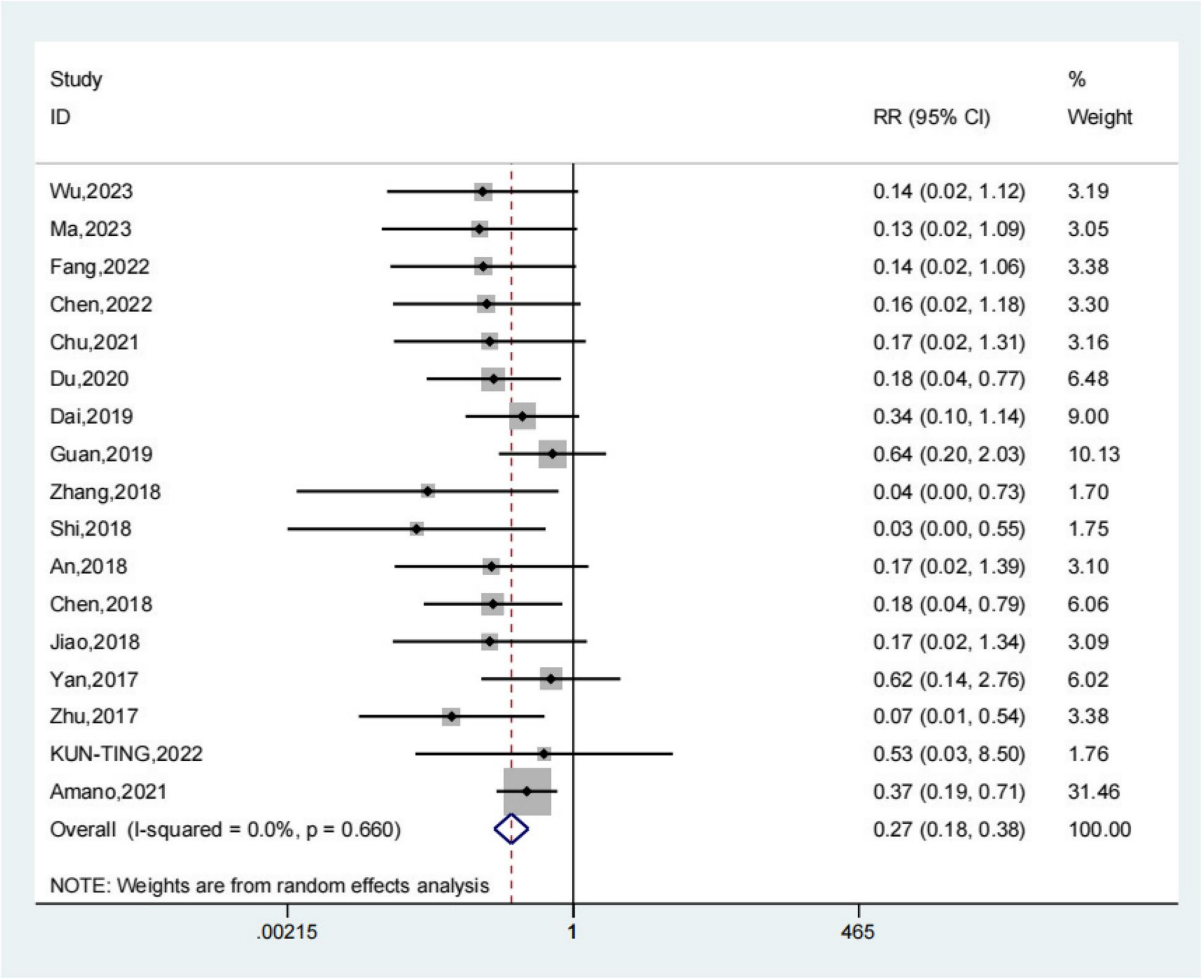
Forest illustration of the recurrence rate

**Fig. 10:**
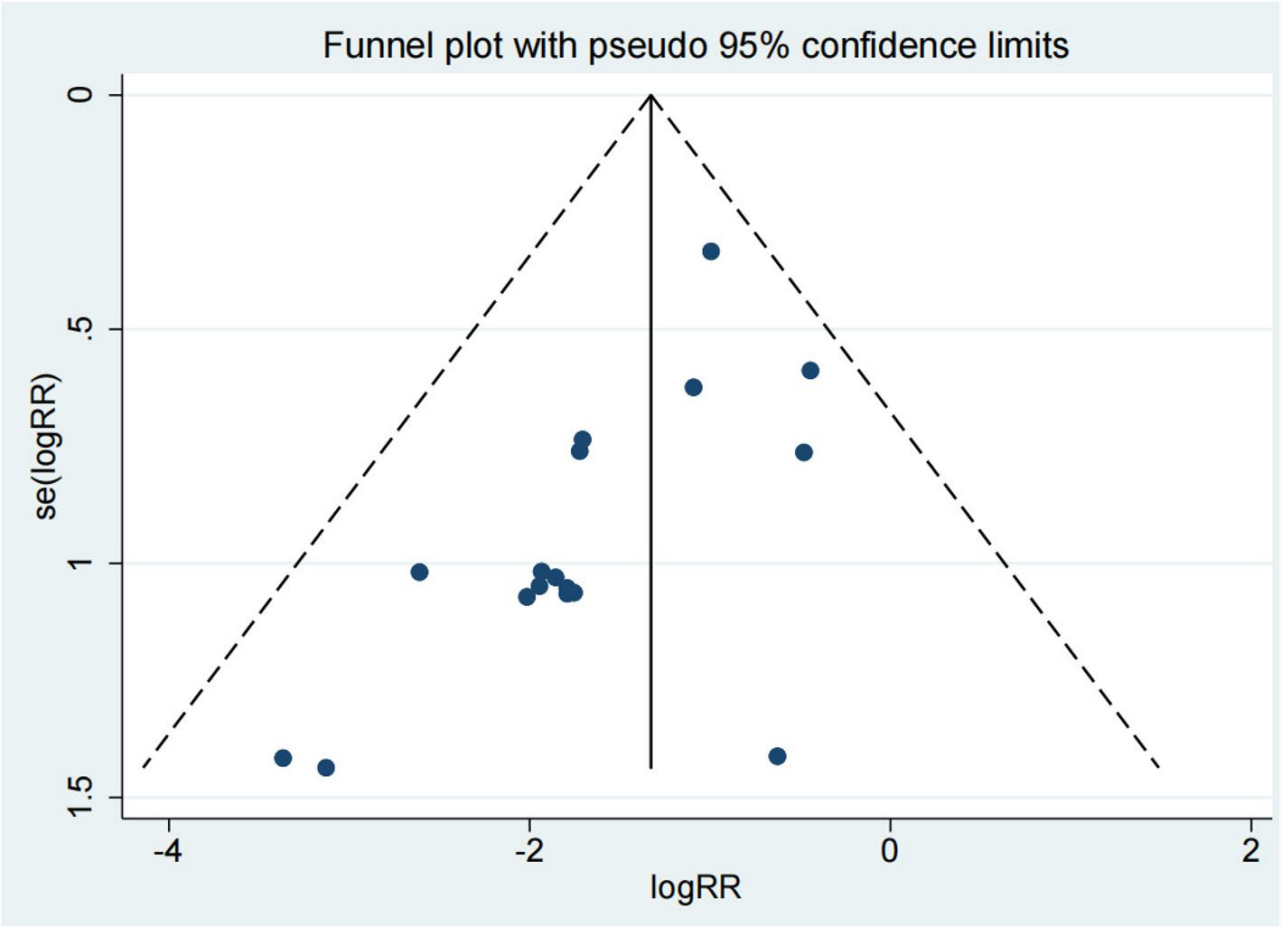
Funnel plot of the recurrence rate

**Fig. 11:**
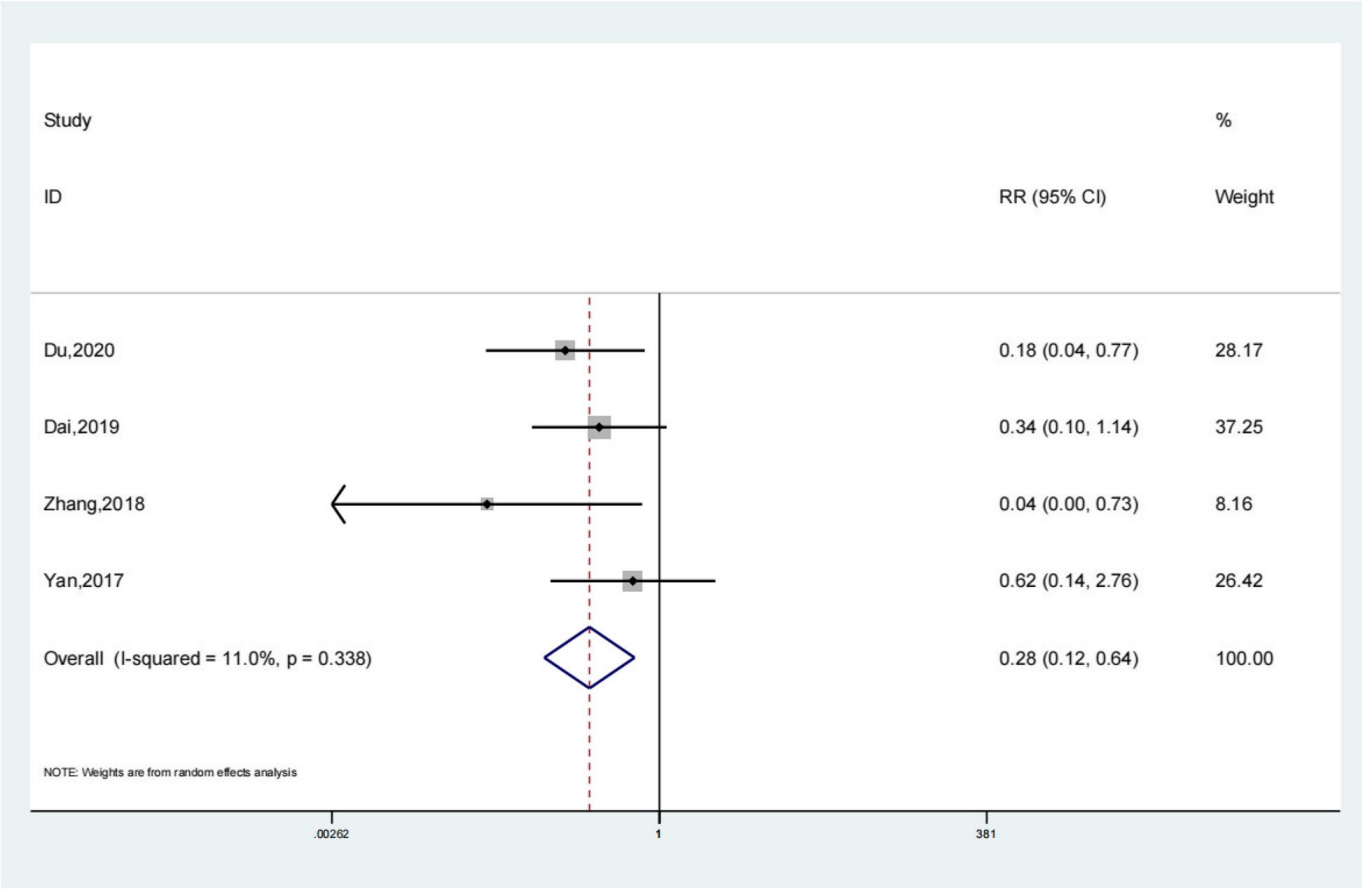
Forest illustration of the recurrence rate of septatus chronic subdural hematoma

Seventeen studies reported the recurrence rate of the test group and the control group. Meta-analysis showed that the recurrence rate of the test group was significantly lower (OR 0.27; 95% Cl 0.18, 0.38; *P* < 0.01, Fig. 9) than the control group. The funnel plot is relatively symmetrical (Fig. 10). Compared with the control group, neuroendoscopy-assisted surgery in the treatment of patients with chronic subdural hematoma decreases the level of recurrence rate. Further analysis was conducted on the recurrence rate of patients with septatus chronic subdural hematoma, and the results showed that patients treated with neuroendoscopy-assisted surgery had relatively less recurrence in patients with septatus chronic subdural hematoma (OR 0.28; 95% Cl 0.12,0.64; *P* < 0.01, Fig. 11).

## Discussion

A chronic subdural hematoma is treated primarily based on its clinical manifestations and cranial imaging findings, which can be divided into surgical treatment and nonsurgical treatment. If the hematoma occupying effect is obvious and causes clinical symptoms, surgical treatment is usually the first choice. Only a small number of patients with chronic subdural hematoma can absorb the intracranial hematoma on their own after non-surgical treatment, surgical treatment mainly includes large or small bone flap craniotomy, drilling and drainage, neuroendoscopic hematoma removal, minimally invasive puncture and drainage, and middle meningeal artery embolization. Surgical treatment is still the most effective treatment method for CSDH patients, because drilling and drainage is a simple and easy-to-apply technique, and drilling and drainage treatment significantly alleviates clinical symptoms. Therefore, drilling and drainage surgery is the most commonly used method for clinical treatment of CSDH [34–36].

Drilling is the main treatment for chronic subdural hematoma. Its complications include intracranial infection, brain tissue injury, intracranial hemorrhage, poor drainage of hematoma, postoperative recurrence, postoperative cerebrospinal fluid leakage, tension pneumocephalus, epilepsy, and postoperative Disorders of consciousness. The recurrence rate of chronic subdural hematoma treated with drilling surgery is 11.7 ~ 12.1%. At present, research has found that closed drainage can significantly reduce the recurrence rate of chronic subdural hematoma, thereby clarifying the necessity of drainage. Level I evidence strongly supports the closure system drainage of the subdural space after drilling, as the recurrence rate and hospitalization time are significantly reduced. Therefore, if feasible, temporary drainage pipes under the dura mater or cap-like aponeurosis should be retained to drain any fluid that accumulates immediately after surgery. Due to the risk of cortical injury, epilepsy, and infection associated with subdural drainage, the cap-like aponeurosis drainage tube is considered a minimally invasive alternative method. Preliminary studies have shown that the recurrence rate and complication rate of the two methods are statistically equivalent. In addition, prospective randomized trials are needed to fully determine whether the efficacy and outcomes of the cap-like aponeurosis drainage tube and the subdural drainage are comparable [37, 38].

For partitioned CSDH, simple skull drilling drainage often leads to residual and recurrent hematoma due to poor drainage, with a recurrence rate of up to 21% reported in the literature. Porous drilling and drainage or repeated operations or even Craniotomy are often required, with large trauma, relatively increased surgical complications, long recovery time, and high treatment costs. Thoroughly removing liquefied and non-liquefied materials under direct vision of neuro endoscopy, and conventional drilling and drainage surgery can only blindly place drainage tubes into the subdural cavity due to the inability to effectively expose and observe hematoma. Sometimes, intraoperative damage may occur due to narrow or hard drainage spaces, such as vascular damage or insertion into brain tissue. It is also difficult to completely remove the hematoma during surgery, so it is necessary to retain the drainage tube, which can lead to a second extubation after surgery and a risk of intracranial infection. Hematoma or flocculent matter and thoroughly clean the hematoma cavity. For partitioned subdural hematomas, direct vision can safely separate and completely remove the contents of each hematoma cavity, achieving the goal of effectively clearing partitioned subdural hematomas. However, due to the inability of rigid endoscopes to bend, attention should be paid to the compliance of instrument operation to reduce surgical complications such as brain contusion and laceration, hematoma, and cerebral infarction. Long-term endoscopic training and experience accumulation are needed to compensate for the shortcomings. In recent years, with the development of neuroendoscopy technology, soft neuroendoscopy has gradually emerged in the treatment of CSDH. Soft neuroendoscopy, due to its advantages such as being able to change direction and angle arbitrarily, having a thin outer diameter, and being flexible in operation, is more suitable for imaging separated subdural hematomas.

A total of 20 literatures were included in this study, including 898 patients in the experimental group and 1448 patients in the control group. Meta-analysis showed that patients with chronic subdural hematoma who received neuroendoscopy-assisted surgery had a lower level of recurrence rate than controls. Meta-analysis showed a satisfactory Recurrence rate for the experimental group (OR 0.27; 95% Cl 0.18, 0.38; *P* < 0.01) and patients treated with neuroendoscopy-assisted surgery had relatively less recurrence in patients with septatus chronic subdural hematoma (OR 0.28; 95% Cl 0.12, 0.64; *P* < 0.01). Based on the results of the meta-analysis of recovery rate, compared to the control group, meta-analysis showed that the recovery rate of the test group was significantly higher (OR 1.18; 95% Cl 1.01, 1.38; *P* = 0.03). Based on the results of the meta-analysis of the total effective rate, compared to the control group, a meta-analysis showed that the total effective rate of the test group was significantly higher (OR 1.11; 95% Cl 1.04, 1.17; *P* < 0.01). Based on the results of the meta-analysis of operative time, compared to the control group, a meta-analysis showed that the operative time of the test group was significantly higher (SMD 15.78; 95% Cl 9.69, 21.86; *P* < 0.01). Meta-analysis showed that the hospital stay of the experimental group was significantly lower (SMD − 1.66; 95% Cl − 2.17, − 1.14; *P* < 0.01). For the results of the meta-analysis of complications, compared with the control group, a meta-analysis showed that the complications of the test group were significantly lower (OR 0.48; 95% Cl 0.30, 0.78; *P* < 0.01).

## Limitations

There are several limitations to this systematic review: it only searched Chinese and English literature, and no other languages were searched, and bias in the selection of studies may have been present. Therefore, you should be objective about some of the results of this meta-analysis.

## Conclusion

In this study, neuroendoscopy-assisted surgery was effective in treating chronic subdural hematoma, as evidenced by a low recurrence rate, a high recovery rate, a high total effective rate, a short operation time, a short hospital stay and few complications, and the above conclusions need to be verified by more high-quality studies.

## Data Availability

Not applicable.
